# First evidence for glial pathology in late life minor depression: S100B is increased in males with minor depression

**DOI:** 10.3389/fncel.2015.00406

**Published:** 2015-10-09

**Authors:** Maryna Polyakova, Christian Sander, Katrin Arelin, Leonie Lampe, Tobias Luck, Melanie Luppa, Jürgen Kratzsch, Karl-Titus Hoffmann, Steffi Riedel-Heller, Arno Villringer, Peter Schoenknecht, Matthias L. Schroeter

**Affiliations:** ^1^Department of Neurology, Max Planck Institute for Human Cognitive and Brain SciencesLeipzig, Germany; ^2^University Clinic for Psychiatry and Psychotherapy, Leipzig UniversityLeipzig, Germany; ^3^LIFE—Leipzig Rsearch Center for Civilization Diseases, Leipzig UniversityLeipzig, Germany; ^4^Institute of Social Medicine, Occupational Health and Public Health (ISAP), Leipzig UniversityLeipzig, Germany; ^5^Institute of Laboratory Medicine, Clinical Chemistry and Molecular Diagnostics, Leipzig UniversityLeipzig, Germany; ^6^Department of Neuroradiology, Leipzig UniversityLeipzig, Germany; ^7^Clinic for Cognitive Neurology, University of LeipzigLeipzig, Germany

**Keywords:** minor depression, late life depression, S100B, BDNF, NSE, glia, white matter hyperintensities, biomarker

## Abstract

Minor depression is diagnosed when a patient suffers from 2 to 4 depressive symptoms for at least 2 weeks. Though minor depression is a widespread phenomenon, its pathophysiology has hardly been studied. To get a first insight into the pathophysiological mechanisms underlying this disorder we assessed serum levels of biomarkers for plasticity, glial and neuronal function: brain-derived neurotrophic factor (BDNF), S100B and neuron specific enolase (NSE). 27 subjects with minor depressive episode and 82 healthy subjects over 60 years of age were selected from the database of the Leipzig population-based study of civilization diseases (LIFE). Serum levels of BDNF, S100B and NSE were compared between groups, and correlated with age, body-mass index (BMI), and degree of white matter hyperintensities (score on Fazekas scale). S100B was significantly increased in males with minor depression in comparison to healthy males, whereas other biomarkers did not differ between groups (*p* = 0.10–0.66). NSE correlated with Fazekas score in patients with minor depression (*r_s_* = 0.436, *p* = 0.048) and in the whole sample (*r_s_* = 0.252, *p* = 0.019). S100B correlated with BMI (*r_s_* = 0.246, *p* = 0.031) and with age in healthy subjects (*r_s_* = 0.345, *p* = 0.002). Increased S100B in males with minor depression, without alterations in BDNF and NSE, supports the glial hypothesis of depression. Correlation between white matter hyperintensities and NSE underscores the vascular hypothesis of late life depression.

## Introduction

Minor depression is a widespread phenomenon in late life (Hegerl and Schoenknecht, 2009; Polyakova et al., 2015). According to the fourth edition of the diagnostic statistical manual of mental disorders (DSM-IV) a person suffering from two to four depressive symptoms for at least 2 weeks has a minor depressive episode. For diagnosis of minor depressive disorder one additionally has to exclude a history of major depression (American Psychiatric Association, 2000). In clinical practice patients with minor depressive symptoms may represent an independent minor depressive episode or a subsyndromal stage of major depression (Park et al., 2010). Every fourth patient with minor depression develops major depression within 2 years after diagnosis (Lyness et al., 1999) and 13% of subjects with minor depression have attempted suicide at least once (Eaton et al., 1995). With regard to these data proper diagnosis and management of minor depression might become an approach to prevent a more severe depressive disorder.

Although plenty of studies have been conducted to elucidate the etiology of major depression, the pathophysiology of minor depression is still unknown. Possible research directions include the glial, neurotrophic and vascular hypotheses of depression. Alterations of peripheral biomarkers of brain structure and function might shed light on the pathological changes in central mechanisms. Brain derived neurotrophic factor (BDNF), S100B and neuron specific enolase (NSE) are among the most studied biomarkers in affective disorders, in particular major depressive disorder (Schroeter et al., 2002; Hetzel et al., 2005; Andreazza et al., 2007; Schroeter and Steiner, 2009; Kalia and Silva, 2015).

BDNF, associated with plasticity in the central and peripheral nervous system, is decreased in serum in acute major depressive episodes and restored in remission (Molendijk et al., 2014). The glial marker protein S100B is elevated during major depressive episodes and decreased following successful treatment (Schroeter et al., 2013). Thus, fluctuations in serum levels of BDNF and S100B seem to be state markers for major depression. This is supported by powerful meta-analyses including a very high number of subjects (Schroeter et al., 2008; Polyakova et al., 2015). NSE is a marker for neuronal injury. In contrast to BDNF and S100B, serum NSE levels seem to be stable in depression suggesting mainly glial dysfunction (Schroeter et al., 2013). However, a recent publication reported increased NSE levels in cerebrospinal fluid (Schmidt et al., 2015), leaving more space for discussion.

Due to clinical similarities with major depression, we expect similar biomarker changes in minor depression. Since BDNF levels do not correlate with depression severity (Molendijk et al., 2011), decreased serum BDNF might also be observed in minor depression. In this disorder it might reflect impaired constitutive or activity-dependent BDNF expression, resulting in impaired brain plasticity. Increased S100B in minor depression may indicate early glial pathology that precedes specific neuronal changes such as in major depression (Rajkowska, 2000). Unaltered (comparing to healthy controls) NSE should confirm that there is no neuronal damage in minor depression.

To further explore the etiology of minor depression we analyzed serum levels of BDNF, S100B and NSE in subjects with minor depression and healthy control subjects from the Leipzig population-based study of civilization diseases (LIFE). Serum levels of BDNF, S100B and NSE were considered as primary outcomes. In analogy to major depression we hypothesized a decrease of BDNF and an increase of S100B, without changes of NSE in minor depression.

An association between late life minor depression and the vascular hypothesis of depression (Taylor et al., 2013) was investigated in explorative analyses by correlating white matter hyperintensities to serum markers. In order to control for confounding variables we correlated age and body mass index (BMI) with serum markers. Correlation of serum markers with clinical and imaging parameters, such as age, BMI and extent of white matter hyperintensities were considered as secondary outcomes.

## Materials and Methods

### Participants

Twenty seven subjects 60 years and older satisfying the DSM-IV criteria for minor depressive episode and eighty two healthy control subjects were selected from the LIFE study. LIFE study includes a representative sample from the Leipzig population (Loeffler et al., 2015). All of the participants provided their written informed consent in accordance with the Declaration of Helsinki before participation in the study. The study was approved by the ethics board of the Medical Faculty of the University of Leipzig. At the moment of subjects’ selection the LIFE study database included 1617 subjects over 60 years of age. Every subject underwent structured psychiatric interview, neuropsychological testing, clinical examination, blood sampling and scanning with multimodal magnetic resonance imaging (MRI).

### Diagnostic Criteria and Laboratory Procedures

Minor depressive episode according to DSM-IV criteria was diagnosed based on the structured clinical interview for DSM-IV axis I disorders (SCID). White matter hyperintensities were rated by experienced neuroradiologists using the Fazekas scale (Fazekas et al., 1987) in fast fluid-attenuated inversion recovery (FLAIR) images.

Blood samples were collected from the subject’s cubital vein at the first day of the study. The mean time between blood sampling and psychiatric interview was 10.0 (4.3) days for subjects with minor depression and 13.0 (9.0) for healthy subjects. All samples were collected uniformly in the morning, following overnight fasting. Serum was prepared using the standard operating procedures. In brief, samples were left for 45 min for clotting, followed by a centrifugation step (10 min, 2750 g, 15°C). Samples were then filled in straws (CryoBioSytems IMV, France) by an automatic aliquoting system (DIVA, CryoBioSytems IMV, France). After that serum samples were stored at −80°C. To minimize freeze-thaw cycles, samples were sorted in a cryogenic work bench (temperatures below −100°C) and automatically stored in tanks with a coolable top frame in the gas phase of liquid nitrogen (Askion, Germany; Loeffler et al., 2015).

S100B and NSE were measured with monoclonal 2-site immunoluminometric assays performed on the fully mechanized system LIAISON (Diasorin, Dietzenbach, Germany). The detection limit for the assays was 0.02 μg/l and 0.04 μg/l (described in detail elsewhere (Streitbuerger et al., 2012). BDNF was measured in serum with an ELISA manufactured by R&D systems (Wiesbaden, Germany). The sensitivity of the assay was 20 ng/L leading to a measuring range of 62.5 until 4000 ng/L. Interassay coefficients of variation were between 9.4 and 11.1% for mean BDNF concentrations between 362 and 2079 ng/L. Note that serum samples were diluted 1:20 before measuring them with the assay.

### Statistical Analyses

Statistical analyses were performed using SPSS version 22 (IBM, Chicago, IL, USA). Complex assessment of the data distributions were performed including visual assessment of the histograms, skewness and kurtosis of the data, as well as by Kolmogorov–Smirnoff and Shapiro-Wilk test. Since the protein levels were non-normally distributed and different numbers of subjects were included in patients’ and controls’ groups we applied non-parametric Mann-Whitney U test for evaluation of group differences. The differences in demographic factors were assessed by independent *t*-test or by chi-square test. The impact of sex differences and a history of depression were assessed by subgroup analyses. The correlation analyses between serum markers, clinical/imaging and demographic data were performed by calculating Spearmen correlation coefficients. We expected directed changes for BDNF and S100B in minor depression in comparison with control subjects, therefore one-tailed α-level for statistical significance was set at 0.05 for these biomarkers. For NSE and the correlation analyses two-tailed α-level at 0.05 was chosen. The statistical power was calculated using G-power 3.1.9.2. (Faul et al., 2009). Generally, data are presented as means and standard deviations (SD). Dot plots represent the distributions of the protein levels and their medians.

## Results

The demographics, clinical and imaging data, and serum marker levels of the patients and healthy control subjects are presented in Table 1. Both cohorts were matched for age, education, BMI and the extent of white matter hyperintensities as measured with the Fazekas scale.

**Table 1:** **Demographical, clinical/imaging data and serum markers in patients and healthy control subjects**.

	Whole group		Males		Females	
	MinD	HC	MinD	HC	MinD	HC
Number (with a history of depression)	27 (14)	82	7 (3)	58	21 (11)	24
Age	71.2 (4.5)	70.0 (4.1)	71.4 (4.8)	70.3 (4.1)	71.1 (4.5)	69.6 (4.3)
Sex (male/female)	7/21***	51/31***
Education (<12years/>12 years)	24/4	58/24	5/2	37/15	19/2	22/9
Fazekas score (0/1/2/3)	6/16/5/0	25/45/12/0	2/4/0/0	17/27/7/0*	4/12/5/0	8/18/5/0
BMI (kg/m^2^)	27.1 (4.9)	28.1 (4.6)	26.7 (2.1)	27.8 (3.7)	27.2 (5.7)	28.7 (5.9)
BDNF (μg/L)	25.8 (5.4)	25.2 (5.9)	29.6 (14.2)	24.7 (4.3)	26.1 (4.9)	26.1 (7.8)
NSE (μg/L)	11.8 (2.6)	11.9 (2.1)	11.9 (2.9)	11.7 (2.3)	11.7 (2.6)	12.2 (1.7)
S100B (μg/L)	0.088 (0.043)	0.086 (0.11)	0.088 (0.03)*	0.067 (0.03)*^†^	0.088 (0.05)	0.12 (0.16)^†^

MinD, minor depression; HC, healthy controls; BMI, body-mass index; BDNF, brain derived neurotrophic factor; NSE, neuron specific enolase; ^†^Significant difference between males and females at *p* < 0.05; *Significant difference between minor depression and healthy controls group at *p* < 0.05; ***Significant difference between minor depression and healthy controls group at *p* < 0.001; Student’s t-test for age and body mass index, chi-square test for categorical data, Mann-Whitney U test for BDNF, NSE, S100B.

**Figure 1:**
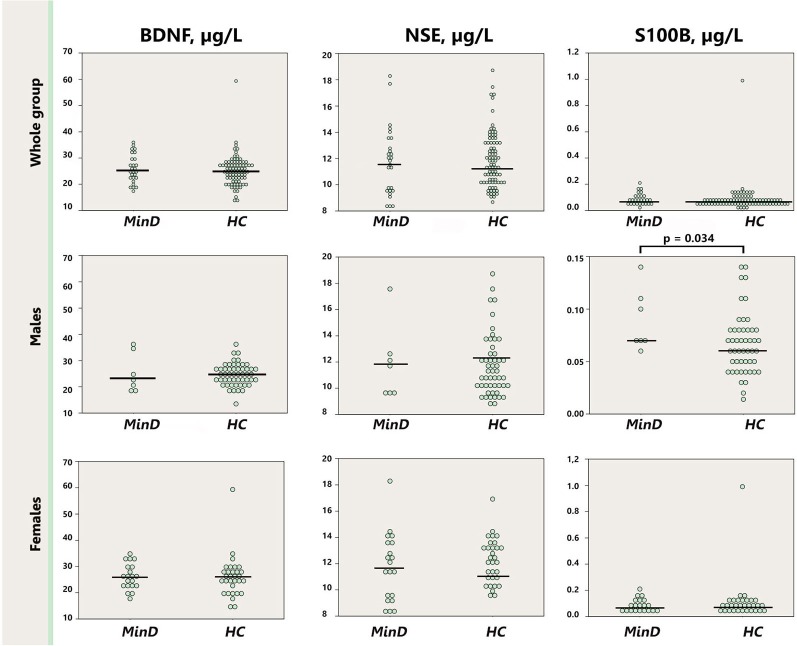
**Dot plot for the distribution of serum markers’ levels in subjects and healthy controls (first row), separately for males (second row), and females (third row).** Median levels of the serum markers are depicted with black horizontal lines. Note that the distribution of S100B in males is depicted on a zoomed scale. MinD, minor depression; HC, healthy controls; BDNF, brain derived neurotrophic factor; NSE, neuron specific enolase.

BDNF, S100B and NSE did not differ between patients in minor depressive episode and healthy control subjects (Table 1). Since the two groups differed with regard to sex, we conducted additionally sex-specific analyses. Figure 1 illustrates results with dot plots for the three serum marker proteins across the whole groups, and specifically for each sex. When the analysis was stratified by sex we observed significantly increased S100B (*p* = 0.034) in males with minor depressive episode (0.092 μg/l [0.012]) in comparison with healthy male controls (0.067 μg/l [0.004]).

**Figure 2:**
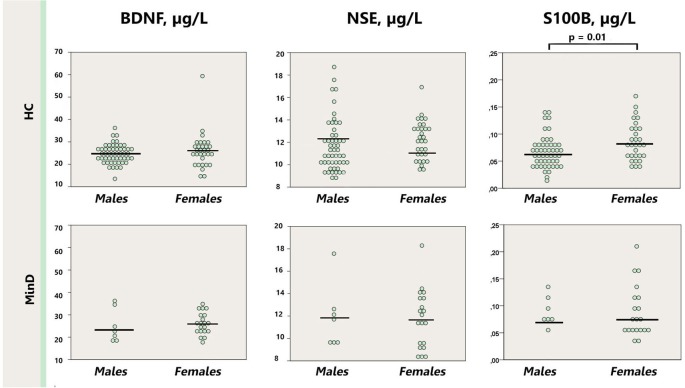
**Dot plot for the distribution of serum markers’ levels in healthy males and females (first row), and males and females with minor depression (third row).** Median levels of the serum markers are depicted with black horizontal lines. Note that the outlier from the healthy females group is not depicted on the S100B plot. MinD, minor depression; HC, healthy controls; BDNF, brain derived neurotrophic factor; NSE, neuron specific enolase.

As depicted in the Figure 2, serum S100B levels were significantly lower in healthy males (0.067 μg/l [0.004]) in comparison with healthy females (0.115 μg/l [0.029]; *p* = 0.01), whereas there was no sex difference in the minor depression group (male: 0.091 μg/l [0.12]; female: 0.088 μg/l [0.011]; *p* = 0.53). Removal the abovementioned female outlier did not affect the differences between healthy males and females for S100B. BDNF and NSE did not differ neither between the groups stratified by sex (males *p* = 0.13–0.95; females *p* = 0.40–0.42), nor when male and female subjects were compared within the minor depression subgroup (*p* = 0.10–0.98).

**Figure 3:**
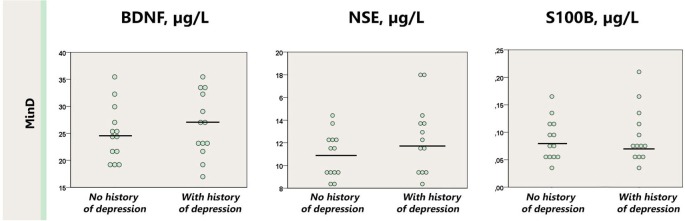
**Dot plot for the distribution of serum markers’ levels in subjects with minor depression with or without a history of depression.** Median levels of the serum markers are depicted with black horizontal lines. MinD, minor depression, HC, healthy controls; BDNF, brain derived neurotrophic factor; NSE, neuron specific enolase.

Similarly, presence of the history of major depression did not affect the levels of BDNF, S100B or NSE in the minor depression group (*p* = 0.10–0.50); neither in comparison with healthy controls (*p* = 0.13–0.38; Figure 3).

As presented on Figure 1 one female control subject presented with extremely high S100B value, above three SD of the group. To control for the impact of this subject on the analysis of S100B we performed an additional analysis of S100B excluding this subject’s data. In this analysis we observed a trend, *p* = 0.078, for increased S100B in the whole minor depression group (0.088 μg/l [0.043]) in comparison with healthy controls (0.074 μg/l [0.032]).

We observed a positive correlation between S100B and BMI in the whole sample (*r_s_* = 0.204, *p* = 0.04), and in healthy subjects (*r_s_* = 0.246, *p* = 0.03), and a positive correlation between S100B and age in the whole sample (*r_s_* = 0.229, *p* = 0.02) and in healthy control subjects (*r_s_* = 0.345, *p* = 0.002).

A significant positive correlation was found between age and the degree of white matter hyperintensities as measured with the Fazekas score both, in the whole sample (*r_s_* = 0.425, *p* < 0.001), and in subgroups (minor depression: *r_s_* = 0.462, *p* = 0.04; healthy controls: *r_s_* = 0.421, *p* < 0.001). With regard to serum markers, Fazekas score correlated positively with NSE in the whole sample (*r_s_* = 0.252, *p* = 0.02) and in patients with minor depression (*r_s_* = 0.436, *p* = 0.048). In the healthy control sample the Fazekas score correlated positively only with S100B (*r_s_* = 0.261, *p* = 0.04).

Finally, we examined whether our groups were large enough to detect the predicted impact of minor depression on serum BDNF and S100B. The statistical power calculations using G-Power for Mann-Whitney tests were based on the previous meta-analyses of BDNF and S100B alterations in major depression (Schroeter et al., 2013; Polyakova et al., 2015). These calculations lead to required sample sizes of *n* = 36 per group for BDNF and *n* = 5 per group for S100B.

## Discussion

In this study, for the first time to our knowledge, we evaluated serum levels of BDNF, S100B and NSE in subjects with minor depressive episode. We found evidence for increased S100B in males with minor depression without any alterations of NSE, which was in agreement with our hypotheses. BDNF was unchanged, although we expected a decrease in analogy to major depression. In assessment of the secondary outcomes we observed a positive correlation between NSE and Fazekas score in minor depression and in the whole sample. S100B correlated positively with age and BMI in the whole sample and in healthy controls.

Our hypotheses were initially built on data derived from major depression studies. In minor depression we didn’t detect the hypothesized difference for BDNF. One explanation of such a negative finding might be that neurotrophic functions are not impaired at the subthreshold level of depression. Then the substantial differences in the pathophysiology of these disorders arise. Nevertheless, one might also argue that the sample size was too low. The calculation of the required sample size using G-Power for BDNF indeed showed that our minor depressive group might have been underpowered (27 subjects instead of the 36 required)_._ In this study we reached only 75% of statistical power. To solve the power issue future studies should involve larger sample size.

For S100B the sample size was obviously large enough to detect the expected group effects (27 subjects instead of five required). Indeed, we observed a trend for increased S100B in the whole minor depression group and significantly increased S100B in males with minor depression in comparison with control subjects. Though we did not rule out potential non-brain sources of S100B in our study, this finding points to the similarities between major and minor depression. Moreover, the fact that differences in S100B are less prominent than in major depression suggests that clinical presentation mirrors to some extend molecular changes.

The findings we describe are based on the concept that serum S100B changes are related to alterations in the brain. However, S100B, as well as BDNF, and NSE might originate from non-brain sources. For instance, various subtypes of leukocytes can secrete S100B (Miki et al., 2013; Fujiya et al., 2014; Moutsatsou et al., 2014). Thrombocytes are the largest source for serum BDNF (Fujimura et al., 2002), adipocytes produce both S100B and BDNF (Fujiya et al., 2014; Huang et al., 2014), finally NSE may originate from damaged peripheral nerves (Li et al., 2013). Because we did not assume relevant biases related to these potentially confounding sources in minor depression, we did not control for potential non-brain sources of the serum markers in our study. Note that we compared subjects with minor depression to matched healthy control subjects and considered, therefore, differences and not absolute values of S100B. Moreover, changes of S100B in leukocytes have been shown only in bipolar disorder (Moutsatsou et al., 2014), whereas for unipolar depression, which is more relevant for our data, no studies are available in the literature so far. Future studies might transcend these limitations by including larger and more strictly selected cohorts and controlling for non-brain sources of these markers.

The concept of leakage from the brain obviously has its limitations. S100B, as well as BDNF, and NSE might originate from non-brain sources. Various subtypes of leucocytes can secrete S100B (Miki et al., 2013; Fujiya et al., 2014; Moutsatsou et al., 2014), thrombocytes are the largest source for serum BDNF (Fujimura et al., 2002), adipocytes produce both S100B and BDNF (Fujiya et al., 2014; Huang et al., 2014), finally NSE originate from damaged peripheral nerves (Li et al., 2013).

Interestingly, S100B was not different between males and females with minor depression, rather it differed between healthy males and females. This finding is in line with previous studies showing no sex differences in major depression (Arolt et al., 2002; Hetzel et al., 2005), but contradicts another one showing increased S100B in females with major depression (Yang et al., 2008). The differences between our and the former study might be attributed to the differences in the studied samples with regard to disease severity and age. Further studies are in agreement with our finding for healthy subjects by showing higher S100B in healthy female than male adults (Streitbuerger et al., 2012) and children (Gazzolo et al., 2003). Overall, the literature on effects of gender on S100B did not reach consensus so far. Whether gender differences in S100B reflect the differences in susceptibility to disease and whether S100B is a gender-specific marker of minor depression needs more systematic assessment.

S100B, as an index for glial alterations, is modified by age in major depression (Schroeter et al., 2011). In minor depression we did not find a correlation between S100B and age. Instead, S100B correlated positively with age in healthy controls. This finding is in line with cerebrospinal fluid studies (van Engelen et al., 1992; Nygaard et al., 1997), but contradicts later serum studies (Wiesmann et al., 1998; Portela et al., 2002). One potential reason for these differences is again the different disease severity. According to Rajkowska’s observations development of depressive disorder starts with glial alterations and progresses with age (Rajkowska, 2000). If late life minor depression is a subtle manifestation of major disorder, absence of correlation between S100B and age in minor depression might add to Raikowska’s hypothesis.

A weak positive correlation between S100B and BMI was not surprising. S100B is secreted by adipocytes and is involved in the pathogenesis of obesity as shown *in vitro* (Fujiya et al., 2014) and *in vivo* (Buckman et al., 2014). Positive correlation of serum S100B with BMI was previously reported in a combined sample of cognitively intact lean and obese subjects (Steiner et al., 2010a) and in subjects with schizophrenia (Steiner et al., 2010b). In our study the positive correlation in the whole sample was likely driven by the healthy subgroup, with no such association in minor depressive episode. As correlations between S100B and age/BMI were detected only in healthy subjects but not in the minor depression group, and both cohorts were matched regarding mean age and BMI, we assume that age and BMI did not drive the S100B effects in minor depression.

The finding of positive correlation between S100B and white matter hyperintensities in healthy subjects is in agreement with a study by Streitbuerger et al. (2012). These authors reported an association between serum S100B and the diffusion tensor imaging parameters fractional anisotropy and radial diffusivity in white matter tracts in healthy females. From the biological point of view increased secretion of S100B might reflect neuroinflammation that accompanies neuronal damage (Kabadi et al., 2015).

We detected also a positive correlation between serum NSE and Fazekas score in the whole sample and in the minor depression subgroup. NSE, a peripheral marker of neuronal damage, might be either of central (Cheng et al., 2014) or peripheral origin (Li et al., 2013). In major depression a central origin is suggested by the correlation with white matter hyperintensities. Finally, the extent of white matter hyperintensities correlated with age in both cohorts, healthy and minor depressive subjects, which is in line with the literature (Nyquist et al., 2015). In combination with the correlation between white matter hyperintensities and the neuronal injury marker NSE in minor depression, our data might support the vascular hypothesis of late life depression (Taylor et al., 2013; Taylor, 2014).

### Limitations

As already discussed our study was limited by a relatively small sample size, which might have hampered, in particular, the detection of BDNF effects. Another limitation might be the inclusion of patients having a history of depression. Thus, not all patients could be qualified as having minor depressive disorder. We addressed this issue in the subgroup analysis and found that inclusion of the subjects with a history of depression did not affect our results. In fact, such a sample mirrors a real life situation when psychiatrists need to make a clinical judgement and select a treatment strategy. Note, moreover, that our subjects were chosen from a representative population study.

The findings we describe are based on the concept that serum S100B changes are related to alterations in the brain. However, S100B, as well as BDNF, and NSE might originate from non-brain sources. For instance, various subtypes of leukocytes can secrete S100B (Miki et al., 2013; Fujiya et al., 2014; Moutsatsou et al., 2014). Thrombocytes are the largest source for serum BDNF (Fujimura et al., 2002), adipocytes produce both S100B and BDNF (Fujiya et al., 2014; Huang et al., 2014), finally NSE may originate from damaged peripheral nerves (Li et al., 2013). Because we did not assume relevant biases related to these potentially confounding sources in minor depression, we did not control for potential non-brain sources of the serum markers in our study. Note that we compared subjects with minor depression to matched healthy control subjects and considered, therefore, differences and not absolute values of S100B. Moreover, changes of S100B in leukocytes have been shown only in bipolar disorder (Moutsatsou et al., 2014), whereas for unipolar depression, which is more relevant for our data, no studies are available in the literature so far. Future studies might transcend these limitations by including larger and more strictly selected cohorts and controlling for non-brain sources of these markers.

## Conclusion

In this study we made a first attempt to assess serum levels of BDNF, S100B, and NSE in minor depression. We found evidence for increased glial marker S100B in males with minor depression and a similar trend in the whole minor depressive group, but no significant evidence of BDNF and NSE alterations. The positive correlation of NSE with Fazekas score as a measure for white matter hyperintensities in minor depression supports the vascular hypothesis of late life depression.

## Author Contributions

MP, PS, MLS have designed the study, analyzed and interpreted the data, drafted and revised the manuscript content; MP and CS selected the subjects from LIFE study database, JK was responsible for the laboratory detection of the serum markers; LL and KTH were responsible for ratings of white matter hyperintensities, CS, KA, ML, TL, SRH, AV contributed to data acquisition. All authors have critically reviewed the manuscript and approved its final version. All authors agree to be accountable for all aspects of the work in ensuring that questions related to the accuracy or integrity of any part of the work are appropriately investigated and resolved.

## Funding

This study has been supported by the International Max Planck Research School on Neuroscience of Communication (IMPRS NeuroCom; MP), by LIFE—Leipzig Research Center for Civilization Diseases at the University of Leipzig—funded by the European Union, European Regional Development Fund and by the Free State of Saxony within the framework of the excellence initiative (CZ, KA, TL, SRH, AV, PS and MLS), by the German Consortium for Frontotemporal Lobar Degeneration, funded by the German Federal Ministry of Education and Research (MLS), and by the Parkinson’s Disease Foundation (MLS; Grant No. PDF-IRG-1307).

## Conflict of Interest Statement

The authors declare that the research was conducted in the absence of any commercial or financial relationships that could be construed as a potential conflict of interest.

## References

[B1] American Psychiatric Association. (2000). Diagnostic and Statistical Manual of Mental Disorders, 4th Edn, *Text Revision* Washington, DC.

[B2] Andreazza A. C. Cassini C. Rosa A. R. Leite M. C. de Almeida L. M. Nardin P.. (2007). Serum S100B and antioxidant enzymes in bipolar patients. J. Psychiatr. Res. 41, 523–529. 10.1016/j.jpsychires.2006.07.013 16956621

[B3] Arolt V. Peters M. Rothermundt M. (2002). Neuroplasticity in major depression may be indicated by S100B. Eur. Psychiatry 17, 159S–159S. 10.1016/s0924-9338(02)80691-5

[B4] Buckman L. B. Anderson-Baucum E. K. Hasty A. H. Ellacott K. L. (2014). Regulation of S100B in white adipose tissue by obesity in mice. Adipocyte 3, 215–220. 10.4161/adip.28730 25068089 PMC4110099

[B5] Cheng F. Yuan Q. Yang J. Wang W. Liu H. (2014). The prognostic value of serum neuron-specific enolase in traumatic brain injury: systematic review and meta-analysis. PLoS One 9:e106680. 10.1371/journal.pone.0106680 25188406 PMC4154726

[B6] Eaton W. W. Badawi M. Melton B. (1995). Prodromes and precursors–epidemiologic data for primary prevention of disorders with slow onset. Am. J. Psychiatry 152, 967–972. 10.1176/ajp.152.7.967 7793466

[B7] Faul F. Erdfelder E. Buchner A. Lang A.-G. (2009). Statistical power analyses using G*Power 3.1: tests for correlation and regression analyses. Behav. Res. Methods 41, 1149–1160. 10.3758/brm.41.4.1149 19897823

[B8] Fazekas F. Chawluk J. B. Alavi A. Hurtig H. I. Zimmerman R. A. (1987). MR signal abnormalities at 1.5-T in Alzheimer dementia and normal aging. AJR Am. J. Roentgenol. 149, 351–356. 10.2214/ajr.149.2.351 3496763

[B9] Fujimura H. Altar C. A. Chen R. Nakamura T. Nakahashi T. Kambayashi J.. (2002). Brain-derived neurotrophic factor is stored in human platelets and released by agonist stimulation. Thromb. Haemost. 87, 728–734. 12008958

[B10] Fujiya A. Nagasaki H. Seino Y. Okawa T. Kato J. Fukami A.. (2014). The Role of S100B in the interaction between adipocytes and macrophages. Obesity (Silver Spring) 22, 371–379. 10.1002/oby.20532 23804363

[B11] Gazzolo D. Michetti F. Bruschettini M. Marchese N. Lituania M. Mangraviti S.. (2003). Pediatric concentrations of S100B protein in blood: age- and sex-related changes. Clin. Chem. 49, 967–970. 10.1373/49.6.967 12765999

[B120] Hegerl U. Schoenknecht P. (2009). Subdiagnostic depression. Are there treatments with clinically relevant effects? Nervenarzt 80, 532–539. 10.1007/s00115-008-2622-z 19396419

[B12] Hetzel G. Moeller O. Evers S. Erfurth A. Ponath G. Arolt V.. (2005). The astroglial protein S100B and visually evoked event-related potentials before and after antidepressant treatment. Psychopharmacology (Berl) 178, 161–166. 10.1007/s00213-004-1999-z 15316717

[B13] Huang T. Larsen K. T. Ried-Larsen M. Moller N. C. Andersen L. B. (2014). The effects of physical activity and exercise on brain-derived neurotrophic factor in healthy humans: a review. Scand. J. Med. Sci. Sports 24, 1–10. 10.1111/sms.12069 23600729

[B14] Kabadi S. V. Stoica B. A. Zimmer D. B. Afanador L. Duffy K. B. Loane D. J.. (2015). S100B inhibition reduces behavioral and pathologic changes in experimental traumatic brain injury. J. Cereb. Blood Flow Metab. [Epub ahead of print]. 10.1038/jcbfm.2015.165 26154869 PMC4671122

[B15] Kalia M. Silva J. C. E. (2015). Biomarkers of psychiatric diseases: current status and future prospects. Metabolism 64, S11–S15. 10.1016/j.metabol.2014.10.026 25467847

[B16] Li J. Zhang H. Xie M. Yan L. Chen J. Wang H. (2013). NSE, a potential biomarker, is closely connected to diabetic peripheral neuropathy. Diabetes Care 36, 3405–3410. 10.2337/dc13-0590 23846809 PMC3816869

[B17] Loeffler M. Engel C. Ahnert P. Alfermann D. Arelin K. Baber R.. (2015). The LIFE-Adult-Study: objectives and design of a population-based cohort study with 10,000 deeply phenotyped adults in Germany. BMC Public Health 15: 691. 10.1186/s12889-015-1983-z 26197779 PMC4509697

[B18] Lyness J. M. King D. A. Cox C. Yoediono Z. Caine E. D. (1999). The importance of subsyndromal depression in older primary care patients: prevalence and associated functional disability. J. Am. Geriatr. Soc. 47, 647–652. 10.1111/j.1532-5415.1999.tb01584.x 10366161

[B19] Miki Y. Gion Y. Mukae Y. Hayashi A. Sato H. Yoshino T.. (2013). Morphologic, flow cytometric, functional and molecular analyses of S100B positive lymphocytes, unique cytotoxic lymphocytes containing S100B protein. Eur. J. Haematol. 90, 99–110. 10.1111/ejh.12036 23130680

[B20] Molendijk M. L. Bus B. A. Spinhoven P. Penninx B. W. Kenis G. Prickaerts J.. (2011). Serum levels of brain-derived neurotrophic factor in major depressive disorder: state-trait issues, clinical features and pharmacological treatment. Mol. Psychiatry 16, 1088–1095. 10.1038/mp.2010.98 20856249 PMC3220395

[B21] Molendijk M. L. Spinhoven P. Polak M. Bus B. A. Penninx B. W. Elzinga B. M. (2014). Serum BDNF concentrations as peripheral manifestations of depression: evidence from a systematic review and meta-analyses on 179 associations (N=9484). Mol. Psychiatry 19, 791–800. 10.1038/mp.2013.105 23958957

[B22] Moutsatsou P. Tsoporis J. N. Salpeas V. Bei E. Alevizos B. Anagnostara C.. (2014). Peripheral blood lymphocytes from patients with bipolar disorder demonstrate apoptosis and differential regulation of advanced glycation end products and S100B. Clin. Chem. Lab. Med. 52, 999–1007. 10.1515/cclm-2013-0978 24497226

[B23] Nygaard O. Langbakk B. Romner B. (1997). Age- and sex-related changes of S-100 protein concentrations in cerebrospinal fluid and serum in patients with no previous history of neurological disorder. Clin. Chem. 43, 541–543. 10.1016/s0303-8467(97)81581-8 9068602

[B24] Nyquist P. A. Bilgel M. Gottesman R. Yanek L. R. Moy T. F. Becker L. C.. (2015). Age differences in periventricular and deep white matter lesions. Neurobiol. Aging 36, 1653–1658. 10.1016/j.neurobiolaging.2015.01.005 25659858 PMC4380525

[B25] Park J. H. Lee J. J. Lee S. B. Huh Y. Choi E. A. Youn J. C.. (2010). Prevalence of major depressive disorder and minor depressive disorder in an elderly Korean population: Results from the Korean Longitudinal Study on Health and Aging (KLoSHA). J. Affect. Disord. 125, 234–240. 10.1016/j.jad.2010.02.109 20188423

[B26] Polyakova M. Stuke K. Schuemberg K. Mueller K. Schoenknecht P. Schroeter M. L. (2015). BDNF as a biomarker for successful treatment of mood disorders: a systematic & quantitative meta-analysis. J. Affect. Disord. 174, 432–440. 10.1016/j.jad.2014.11.044 25553404

[B27] Portela L. V. Tort A. B. Schaf D. V. Ribeiro L. Nora D. B. Walz R.. (2002). The serum S100B concentration is age dependent. Clin. Chem. 48, 950–952. 12029017

[B28] Rajkowska G. (2000). Postmortem studies in mood disorders indicate altered numbers of neurons and glial cells. Biol. Psychiatry 48, 766–777. 10.1016/s0006-3223(00)00950-1 11063973

[B29] Schmidt F. M. Mergl R. Stach B. Jahn I. Schoenknecht P. (2015). Elevated levels of cerebrospinal fluid neuron-specific enolase (NSE), but not S100B in major depressive disorder. World J. Biol. Psychiatry 16, 106–113. 10.3109/15622975.2014.952776 25264292

[B30] Schroeter M. L. Abdul-Khaliq H. Diefenbacher A. Blasig I. E. (2002). S100B is increased in mood disorders and may be reduced by antidepressive treatment. Neuroreport 13, 1675–1678. 10.1097/00001756-200209160-00021 12352625

[B31] Schroeter M. L. Abdul-Khaliq H. Krebs M. Diefenbacher A. Blasig I. E. (2008). Serum markers support disease-specific glial pathology in major depression. J. Affect. Disord. 111, 271–280. 10.1016/j.jad.2008.03.005 18430474

[B32] Schroeter M. L. Sacher J. Steiner J. Schoenknecht P. Mueller K. (2013). Serum S100B represents a new biomarker for mood disorders. Curr. Drug Targets 14, 1237–1248. 10.2174/13894501113149990014 23701298 PMC3821390

[B33] Schroeter M. L. Steiner J. (2009). Elevated serum levels of the glial marker protein S100B are not specific for schizophrenia or mood disorders. Mol. Psychiatry 14, 235–237. 10.1038/mp.2008.85 19229202

[B34] Schroeter M. L. Steiner J. Mueller K. (2011). Glial pathology is modified by age in mood disorders - a systematic meta-analysis of serum S100B *in vivo* studies. J. Affect. Disord. 134, 32–38. 10.1016/j.jad.2010.11.008 21144594

[B35] Steiner J. Schiltz K. Walter M. Wunderlich M. T. Keilhoff G. Brisch R.. (2010a). S100B serum levels are closely correlated with body mass index: an important caveat in neuropsychiatric research. Psychoneuroendocrinology 35, 321–324. 10.1016/j.psyneuen.2009.07.012 19674846

[B36] Steiner J. Walter M. Guest P. Myint A. M. Schiltz K. Panteli B.. (2010b). Elevated S100B levels in schizophrenia are associated with insulin resistance. Mol. Psychiatry 15, 3–4. 10.1038/mp.2009.87 20029405

[B37] Streitbuerger D.-P. Arelin K. Kratzsch J. Thiery J. Steiner J. Villringer A.. (2012). Validating serum S100B and neuron-specific enolase as biomarkers for the human brain - a combined serum, gene expression and MRI study. PLoS One 7:e43284. 10.1371/journal.pone.0043284 22916238 PMC3422594

[B38] Taylor W. D. (2014). Clinical practice. Depression in the elderly. N. Engl. J. Med. 371, 1228–1236. 10.1056/NEJMcp1402180 25251617

[B39] Taylor W. D. Aizenstein H. J. Alexopoulos G. S. (2013). The vascular depression hypothesis: mechanisms linking vascular disease with depression. Mol. Psychiatry 18, 963–974. 10.1038/mp.2013.20 23439482 PMC3674224

[B40] van Engelen B. G. Lamers K. J. Gabreels F. J. Wevers R. A. Van Geel W. J. Borm G. F. (1992). Age-related changes of neuron-specific enolase, S-100 protein and myelin basic protein concentrations in cerebrospinal fluid. Clin. Chem. 38, 813–816. 1375875

[B41] Wiesmann M. Missler U. Gottmann D. Gehring S. (1998). Plasma S-100b protein concentration in healthy adults is age- and sex-independent. Clin. Chem. 44, 1056–1058. 9590385

[B42] Yang K. Xie G.-R. Hu Y.-Q. Mao F.-Q. Su L.-Y. (2008). The effects of gender and numbers of depressive episodes on serum S100B levels in patients with major depression. J. Neural Transm. 115, 1687–1694. 10.1007/s00702-008-0130-8 18982242

